# User Perspectives on Exergames Designed to Explore the Hemineglected Space for Stroke Patients With Visuospatial Neglect: Usability Study

**DOI:** 10.2196/games.8013

**Published:** 2017-08-25

**Authors:** Bernadette C Tobler-Ammann, Elif Surer, Ruud H Knols, N Alberto Borghese, Eling D de Bruin

**Affiliations:** ^1^ Physiotherapy and Occupational Therapy Research Center Directorate of Research and Education University Hospital Zurich Zurich Switzerland; ^2^ Care and Public Health Research Institute [CAPHRI] Maastricht University Maastricht Netherlands; ^3^ Graduate School of Informatics Department of Modeling and Simulation Middle East Technical University Ankara Turkey; ^4^ Applied Intelligent Systems Laboratory Department of Computer Science Università degli Studi di Milano Milan Italy; ^5^ Institute of Human Movement Sciences and Sport Department of Health Sciences and Technology ETH Zurich Zurich Switzerland

**Keywords:** usability, user perspective, mixed-methods, exergames, visuo-spatial neglect, stroke

## Abstract

**Background:**

Visuospatial neglect due to stroke is characterized by the inability to perceive stimuli emerging in the area opposite to the side of brain damage. Besides adopting conventional rehabilitation methods to treat neglect symptoms, the use of virtual reality (VR) is becoming increasingly popular. We designed a series of 9 exergames aimed to improve exploration of the neglected side of space. When new VR interventions are designed, it is important to assess the usability aspects of such management strategies within the target population. To date, most studies used questionnaires to assess user satisfaction with the intervention or product being tested. However, only a combination of both quantitative and qualitative data allows a full picture of user perspective.

**Objective:**

The purpose of this study was to quantitatively and qualitatively assess patient and therapist perspectives of a VR intervention based on the series of 9 exergames designed to explore hemineglected space. Specifically, we wanted to evaluate (1) perceived-user friendliness of the exergames, (2) attitude towards using the exergames, and (3) intention to use the exergames in the future.

**Methods:**

A total of 19 participants (7 patients, 12 therapists) evaluated the exergames they had used 5 times a week during 3 weeks. The Technology Acceptance Model (TAM) questionnaire was filled out after the intervention. Based on those responses, we conducted focus group interviews (with therapists) and individual interviews (with patients). To analyze the TAM questionnaires, we used descriptive statistics. We adopted content and comparative analysis to analyze the interviews and drew illustration maps to analyze the focus group interviews.

**Results:**

The therapists took a more critical stance with a mean TAM questionnaire total score of 48.6 (SD 4.5) compared to the patients who had a mean total score of 56.1 (SD 12.3). The perceived user-friendliness score was 5.6 (SD 1.4) for patients and 4.9 (SD 1.4) for therapists. The attitude towards using the exergames was rated 4.8 (SD 1.9) by patients and 3.6 (SD 1.4) by therapists, respectively. The intention to use the exergames in the future was rated 3.9 (SD 2.1) by patients and 3.7 (SD 1.8) by therapists. We gained information on how to improve the exergames in the interviews.

**Conclusions:**

Patients and therapists perceived the exergames as user-friendly; however, using the games further with the actual test version was not perceived as conceivable. The therapists were generally more critical towards future use than the patients. Therefore, involving both users to achieve acceptable and user-friendly versions of game-based rehabilitation for the future is deemed crucial and warranted.

**Trial Registration:**

Clinicaltrials.gov NCT02353962; https://clinicaltrials.gov/ct2/show/NCT02353962 (Archived by WebCite at http://www.webcitation.org/6soxIJlAZ)

## Introduction

Stroke-related visuospatial neglect (VSN) due to a right-sided brain lesion (RBL) is characterized by the inability to perceive stimuli emerging in the area opposite to the side of brain damage [1,2]. VSN patients usually have lower scores on disability tests and require longer rehabilitation periods compared to stroke patients without neglect [3,4]. Thus, VSN influences most activities of daily living such as eating, reading, and getting dressed [2,5].

Besides adopting conventional rehabilitation methods to treat stroke-related VSN symptoms, the use of virtual reality (VR) in their assessment and treatment is becoming increasingly popular [6-8]. VR is defined as “an advanced form of human-computer interface that allows the user to ‘interact’ with and become ‘immersed’ in a computer-generated environment in a naturalistic fashion” [9]. Reasons for this increasing popularity might be found in the many advantages attributed to VR, for example, the ability to provide a safe but engaging environment [10], immediate feedback on performance, and repetitive task training with quantifiable continuous progression of training [9]. For example, VR training in isolation or in combination with conventional therapy approaches proved to be superior for the improvement of lower extremity function in stroke patients [11]. However, despite this, evidence for VR therapies being superior to conventional intervention methods for treating VSN is so far somewhat limited [6-8]. Evidence shows that VR has the capacity both to enhance current methods for the assessment and rehabilitation of VSN and to provide new ones. Tsirlin et al [8] presented three major challenges for successful implementation of VR systems in VSN therapy: (1) ergonomic aspects in the sense that mobile, lightweight VR systems are required for rehabilitation, (2) the complexity of VR systems insofar as treating clinic staff do not necessarily have programming skills, and (3) the prohibitive costs of VR devices (eg, for immersive VR systems with head-mounted displays or cyber gloves) [7,8,12]. For these reasons, VR rehabilitation platforms have been mainly restricted to laboratories and to prototypical systems [8] and have not been widely implemented in patients’ homes.

**Figure 1:**
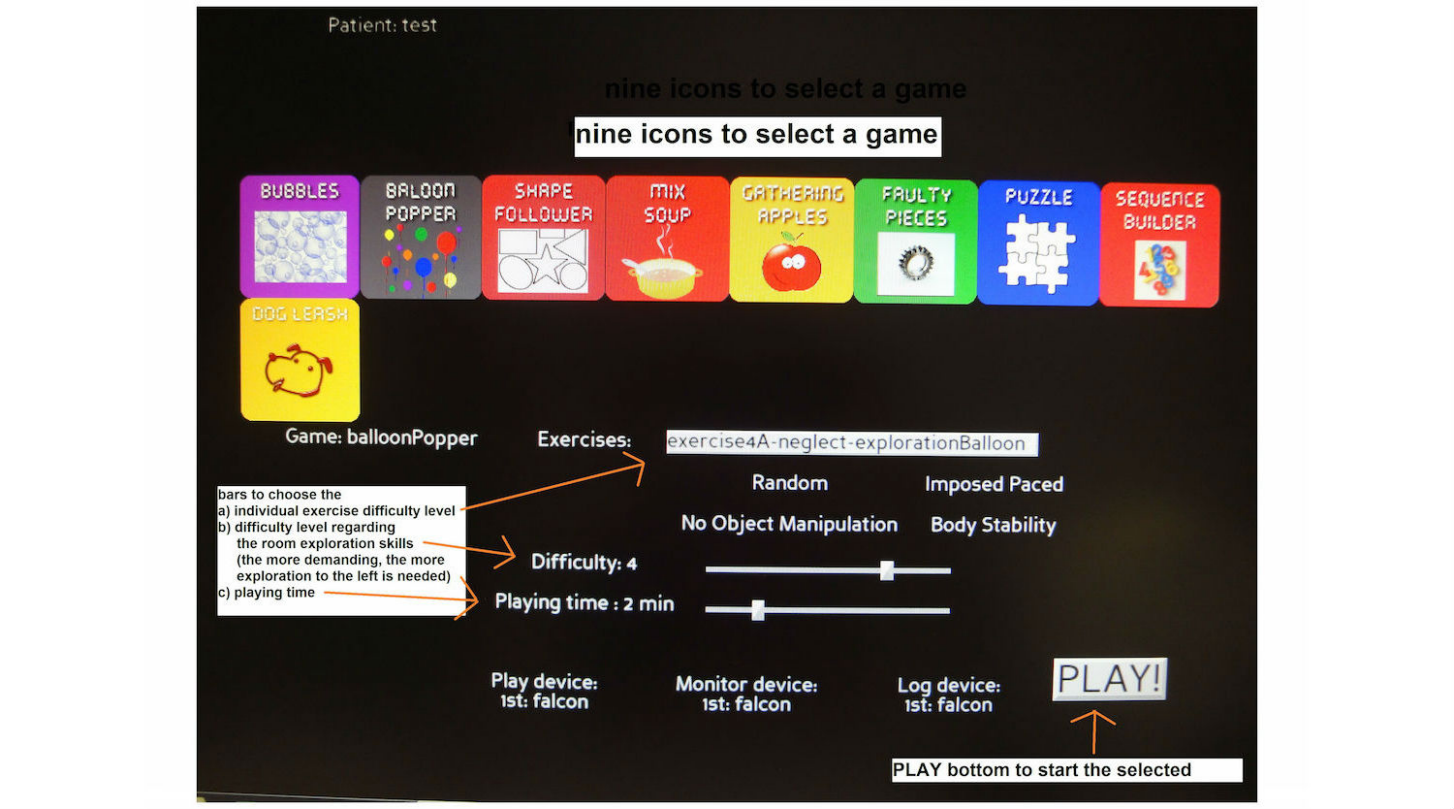
Game menu of the 9 neglect exergames.

**Figure 2:**
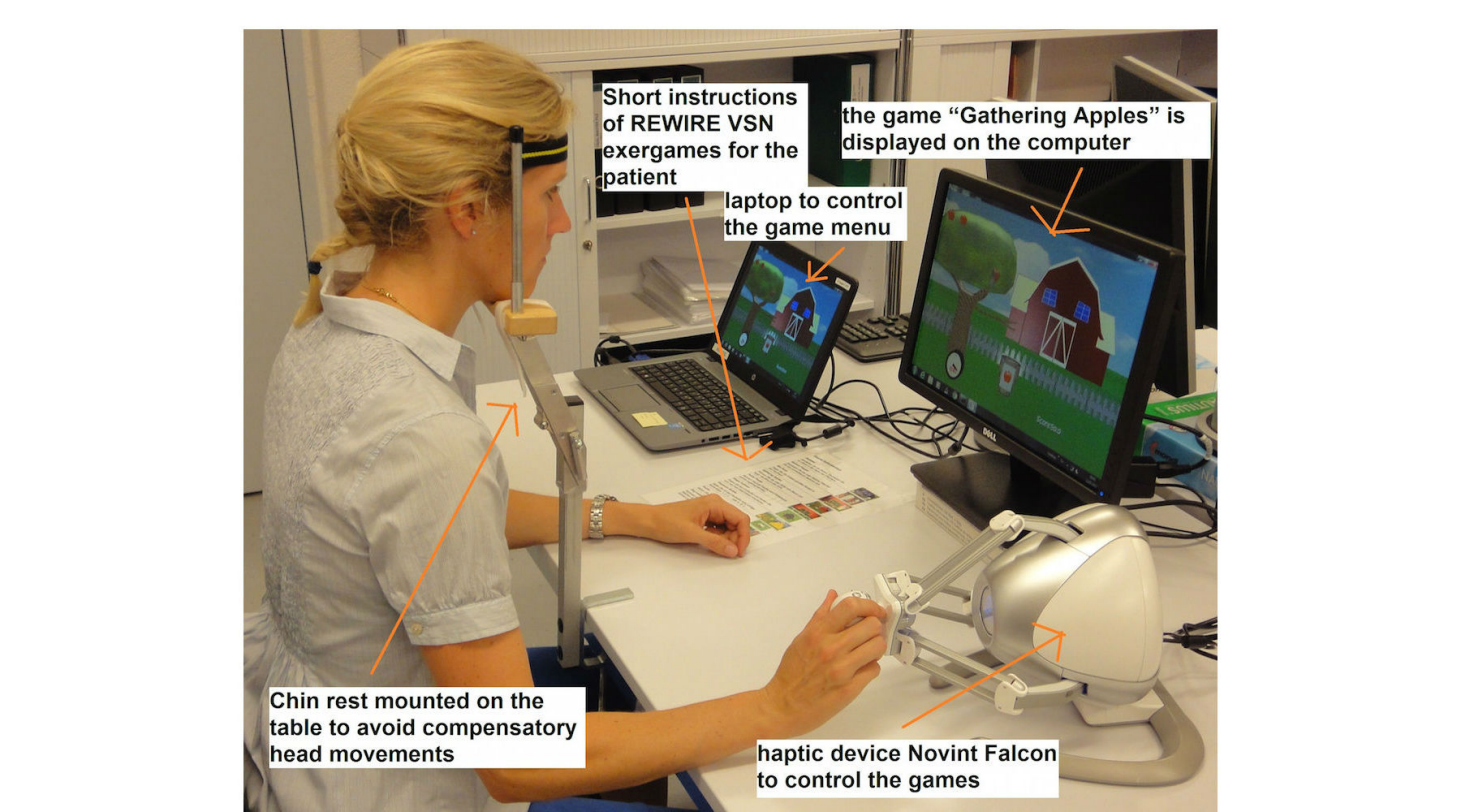
REWIRE exergames training station.

**Figure 3:**
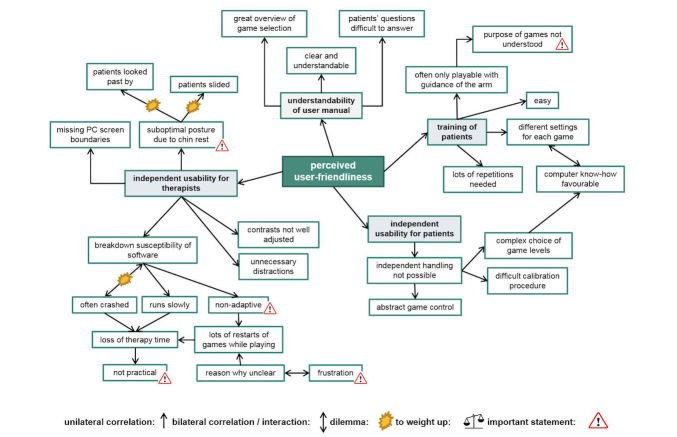
Focus group illustration map: perceived user-friendliness.

A European research group, Rehabilitative Wayout In Responsive Home Environments (REWIRE), developed a game-based VR rehabilitation intervention trying to account for those challenges [13]: (1) the exergame station was designed as a computer workplace, allowing the patient to practice the exergames in a seated position, (2) the complexity of the user interface was reduced to a minimum by designing a game menu with large and clear icons to select a game, difficulty level, and playing time (Figure 1), and (3) the costs of the VR systems are relatively low, as the exergames are played on a personal computer, using the Novint Falcon haptic device (Novint Technologies) to control the games [14] (Figure 2). The Novint Falcon enables people to experience a realistic sense of touch by providing force and haptic feedback when reaching for and grasping virtual objects [14]. Furthermore, it can be operated with one hand only, thus permitting VSN patients to play the exergames with their unaffected upper limb.

When new VR interventions are designed, it is important to follow a phased iterative approach, wherein the usability aspects of such a management strategy, within the target population, are first assessed [15]. Usability is defined by the International Organization for Standardization (ISO) as “the extent to which a product can be used by specified users to achieve specified goals with effectiveness, efficiency and satisfaction in a specified context of use” [16]. “Specified users” do not only include patients but also therapists, as their requirements for the use of such games may differ from those of the patients [17]. Therapists may, for example, need an easy startup and configuration procedure, or stress that the games should be supportive not only for the patient during play but also for the therapist in tracking a patient’s performance [17]. It is therefore important to assess both the therapists’ and patients’ opinions, as they will use the exergames at least as often as a patient.

Assessing opinions from users can be done by using questionnaires or by means of interviews. The former has the advantage to assess many opinions of a representative sample but cannot tell us about the meaning behind a response. The latter is usually applied in a smaller sample but provides personal thoughts from an insider’s perspective [18]. To date, most studies used questionnaires to assess users’ satisfaction with the intervention or product being tested [19-22]. Currently, there is a lack of evidence from studies examining the users’ perspective via application of qualitative methodologies [23]. King et al [24] and Lewis et al [25], for example, used focus group and semistructured interviews to assess patients’ satisfaction with their game intervention. All participants enjoyed playing the computer games. However, all stroke patients were in the chronic stage of recovery and none were diagnosed with VSN symptoms. Another qualitative report explored the perceptions and personal experiences of stroke survivors regarding a leisure-based VR program [26], reporting improved self-efficacy belief in leisure activities after the VR experience in in-depth interviews. However, the reported evidence was based on a single game session only.

Results of a recent systematic review state that in the posttest stage of usability evaluation, performing interviews to evaluate user perceptions of games is recommended, whereas the use of questionnaires is considered useful for evaluating user acceptance and satisfaction [27]. As a consequence, only a combination of both quantitative and qualitative data allows a full picture of user perspectives, as it is done in mixed methods research [18]. Therefore, the purpose of this study was to quantitatively and qualitatively assess the patients’ and therapists’ user perspective when using REWIRE exergames for rehabilitation of VSN symptoms due to a stroke. Specifically, we wanted to evaluate the (1) perceived-user friendliness of the exergames, (2) attitude towards using the exergames, and (3) intention to use the exergames in the future.

## Methods

### Study Design

For this usability study, we used a mixed methods design adopting the “sequential explanatory” design strategy [18]. This design strategy is characterized by an initial collection of quantitative data followed by a collection and analysis of qualitative statements. The purpose of this strategy is to use the qualitative results to assist in explaining and interpreting the findings of the quantitative data.

### Participants

There were 2 groups of users involved in this study: patients as end users and therapists as experts.

The patient group included 7 adults with an ischemic (n=5 men) or hemorrhagic (n=2 women) RBL due to a first stroke with accompanying VSN symptoms as measured with the Catherine Bergego Scale (CBS) [28]. The CBS includes direct observation of the patient’s functioning in 10 real-life situations. The functioning is rated from 0-30, where 0 indicates no neglect symptoms. These patients simultaneously participated in a feasibility study in which the exergames were evaluated, while taking part in this usability evaluation [29]. Their mean age was 68.6 (SD 8.9) years. Their stroke incidence took place 46.3 (SD 30.8) days before study entry. All patients were right-handed. Three participants were able to walk, while the others used a wheelchair for locomotion. Their CBS mean score was 9.4 (SD 5.1) points. All but one of these 7 patients identified themselves as having a computer at home prior to participating in the study.

The expert group consisted of therapists responsible for the treatment of the stroke patients during their inpatient stay. The 12 therapists (6 occupational therapists from one rehabilitation clinic and 6 neuropsychologists from another clinic) supervised and trained the patients in the use of the REWIRE exergames during the 3-week intervention phase. Their mean age was 33.3 (SD 5.7) years (range 27-45) with a mean work experience of 6.8 (SD 5.8) years (range 0.5-20). All therapists stated being familiar with the use of computers, rating their computer knowledge as excellent (n=3), good (n=8), and poor (n=1). All 19 participants signed informed written consent before study entry. We obtained ethical approval for the study from the local Ethics Committees (Zurich No. 2014-0543 and Bern No. 389/2014) as well as from Swissmedic (2015-MD-0003). The study is registered with ClinicalTrials.gov.

### Setup of the Exergames Training Stations

We installed 2 exergames training stations, one in each of the collaborating clinics (Figure 2). The games were played at a table in a seated position either in a chair or wheelchair depending on the patient’s motor skills. We used a 21-inch computer monitor at a distance of 60-65 cm to display the games and the haptic Falcon Novint device to control the games. The haptic feedback enabled the patients to experience a realistic sense of touch, for example, by feeling some resistance simulating the weight of the currently held virtual object in the virtual hand displayed on the screen (force feedback of the Falcon [14]) or a vibration when dropping, for example, a virtual apple in a virtual basket. The Falcon Novint was placed at the side of the computer monitor at a distance allowing the patients ease of reach with their nonaffected hand. A height-adjustable chin rest (Novavision GmbH) was mounted on the table to avoid compensatory head movements while playing the exergames.

### REWIRE Visuospatial Neglect Exergames

We designed a series of 9 exergames aimed to improve exploration of the neglected side of space. During the development of the exergames, we regularly tested them in healthy controls prior to implementing them in a clinical setting. Their feedback was constantly integrated in the development process until consensus was reached. In order to maintain principles of training, game progression was individually adjustable through the selection of appropriate different levels of difficulty (more demanding meant more exploration towards the hemineglected side was required) [30]. The exergames content aimed to imitate activities of daily living (ADL), such as cooking a meal, following a recipe, gathering apples, walking a dog, and doing a puzzle. An overview of the 9 games and corresponding short instructions supporting their independent use are shown in Multimedia Appendix 1. A detailed description of the exergames can be found elsewhere [13].

### Intervention

Both therapists and patients had the opportunity to test the exergames before entering the study, followed by a training event organized by the research team to learn, for example, how to handle the game menu and Falcon Novint haptic device. During the whole intervention phase, the research staff provided telephone or personal support whenever needed, for example, to handle technical problems with the training station. The exergames intervention lasted 3 weeks and included 15 training sessions each of approximately 30 minutes duration. Patients exercised with the games under supervision of the therapists depending on their required level of support, for example, to start a new game. The neuropsychologists included exergames playing in their computer group, meaning that participating patients played the REWIRE exergames while other group members performed alternative computer tasks. The occupational therapists (OTs) supervised their patients in a one-to-one setting during individual therapy sessions. Additionally, the supervising therapist individually adjusted the intensity of playing the exergames. This was done, for example, by changing the difficulty level or game duration in the game menu or by implementing short breaks between each game if needed. Each patient selected up to four REWIRE VSN exergames from the game menu to be played in a gaming session. The choice was based on personal interest of the patient, which was assumed to enhance motivation while playing. Therefore, during the 3-week intervention time, the patient was also allowed to change games if they wanted to test another game or felt bored with the previously played one. However, we suggested the patients test all of the 9 games at least once. Rehabilitation continued during the study intervention, our exergames serving as an additional therapy option to the standard program comprising daily occupational, physical, and neuropsychological therapy.

### Outcome Measurements

Both patients and therapists completed a questionnaire at the end of the intervention. This included 12 questions with a 7-point Likert scale (1 point=strongly disagree; 7 points=strongly agree), evaluating (1) perceived user-friendliness of the exergames, (2) attitude towards using the exergames, and (3) intention to use the exergames in the future. The questionnaire design was based on an abridged version of the Technology Acceptance Model (TAM). TAM is an intention-based model developed specifically for explaining user acceptance of computer technology [31] that we considered useful for evaluating user acceptance and user satisfaction [27]. Patients received physical assistance from clinic staff to complete the questionnaire when incapable of writing or reading due to neglect. The therapists filled in their questionnaires independently. We analyzed the completed questionnaire responses and thereafter used them as a basis to prepare the individual interview [32] with the patients and the focus group interviews [33] with the therapists.

BC-T-A performed the audio-recorded individual interviews during the follow-up assessment planned for the feasibility study 4 weeks post-intervention. They focused on the patients’ everyday life experiences with right hemispheric stroke and VSN symptoms during active rehabilitation and served as an opportunity to deepen, clarify, or confirm answers that were given in the TAM questionnaire. BC-T-A, who is an occupational therapist, took an active role during the interviews, aiming to build a relationship with the participants based on confidence and co-creation. Thanks to the many opportunities to meet the patients in the past (eg, while introducing the exergames to the patients or during data generation for the simultaneously running feasibility study [29]), a good basis to achieve this aim was already established.

We conducted the two focus group interviews in the collaborating rehabilitation clinics after the last patient had finished the REWIRE exergames intervention. We audio-recorded and filmed them. In comparison to individual interviews, focus groups represented group opinions influenced by social interactions and team dynamics, as therapists already knew each other well [34]. Therefore, they were an important complement to individually given answers via TAM questionnaires and served as a means for therapists to gain, share, or dispute experiences made with those exergames from their perspectives. During each interview, the moderator (BC-T-A) summarized the given answers on a flip chart, allowing therapists to add, complement, or change statements if needed. Additionally, the role of the moderator was (1) to balance the therapists’ statements in terms of allowing/encouraging everybody to speak, while intervening when someone would have claimed too much time to speak and (2) to take care that all therapists’ TAM answers were discussed that needed clarification while keeping the time set for the focus group interview.

We expected the acceptance of and satisfaction with the neglect exergame intervention to be good, which we defined as a total mean score of more than 4 points on the Likert scale, where 1-3 points meant no agreement, 4 points neutral, and 5-7 points agreement. The maximum achievable score was 84 points, indicating perfect agreement. For the individual and focus group interviews, we hypothesized that the users (therapists and patients) would have experienced the VR-based neglect training as supportive in treating VSN.

### Data Analysis

To analyze the TAM questionnaires, we used descriptive statistics in SPSS software version 23. We calculated means, standard deviations, medians, and interquartile range values as appropriate.

We transcribed the individual interviews verbatim. Subsequently, we selected text passages from the entire conversation in which the interviewer (BC-T-A) and the patient discussed the use of the exergames and stored them separately. We used content and comparative analysis to analyze those passages [35,36], taking the following analysis steps: (1) reading the interview passages’ transcriptions, (2) highlighting significant statements that provide an understanding of how the patient experienced the use of the exergames, (3) comparing those statements with the TAM questionnaire answers and assigning them to the three subcategories (user-friendliness, attitude, and intention to use in the future), and (4) writing a composite description of the patients’ perspectives of using the exergames, while using quotes to underpin the interpretation.

We analyzed the two focus group interviews by drawing “Focus group Illustration Maps” (FIMs) [33]. The aim was to summarize the complex variety of statements and opinions without losing information or knowledge. Therefore, capturing the whole range of group knowledge is the essence of knowledge mapping, rather than highlighting the single statements of individuals. We took the following analysis steps: (1) listening to the audio recording while watching the video and taking notes, (2) comparing the notes and audio recordings together with the flipchart notes, (3) drawing the FIMs, re-watching the video to check the accuracy of the FIMs, (4) sending the FIMs to the participants for member checking, (5) incorporating feedback from participants into the FIMs if representative for the whole group, and (6) merging FIMs from both clinics into one FIM per subcategory from the TAM questionnaires, representing the opinions from all 12 participating therapists.

## Results

We summarized the answers from the TAM questionnaires in Table 1 for patients and in Table 2 for therapists. Generally, the therapists took a more critical stance with a mean TAM questionnaire total score of 48.6 (SD 4.5) compared to the patients with a mean total score of 56.1 (SD 12.3). Their statements are presented according to the three subcategories of the TAM questionnaire. These are “perceived user-friendliness,” “attitude towards using the exergames,” and “intention to use the exergames in the future.”

**Table 1:** Postintervention patients’ TAM questionnaire responses.

Statement		P1^a^	P2	P3	P4	P5	P6	P7	Mean (SD)	Median^b^ (Q_1_/Q_3_)	Mean (SD)
**Perceived user-friendliness**											
	The exergames were easy to use.	5	6	7	7	7	6	5	6.1 (0.9)	6 (5/7)	5.6 (1.4)
	The exergames manual was clear and understandable.	5	6	7	5	7	7	6	6.1 (0.9)	6 (5/7)	
	Learning to use the exergames independently would be easy for me.	3	5	6	4	7	2	5	4.6 (1.7)	5 (3/6)	
**Attitude towards using the exergames**											
	I generally have a positive attitude towards using the exergames.	5	7	2	6	7	1	6	4.9 (2.4)	6 (2/7)	4.8 (1.9)
	I enjoyed exercising with the exergames.	4	6	2	6	7	5	3	4.7 (1.8)	5 (3/6)	
**Exercising with the exergames…^c^**											
	was motivating.	4	6	2	7	7	4	5	5.0 (1.8)	5 (4/7)	
	was exhausting.	3	7	1	3	4	7	2	3.9 (2.3)	3 (2/7)	
	was a stupid idea.	5	7	4	6	7	5	4	5.4 (1.3)	5 (4/7)	
**Intention to use the exergames in the future: If I had access to the exergames from at home, …**											
	I would use them in the future.	5	5	1	4	1	4	1	3.0 (1.9)	4 (1/5)	3.9 (2.1)
	I would use them regularly.	4	5	1	4	1	3	1	2.7 (1.7)	3 (1/4)	
	I’m convinced that my family/friends would support me using the exergames.	5	3	2	6	7	4	6	4.7 (1.8)	5 (3/6)	
	I would recommend the exergames to other patients.	6	7	1	6	7	2	6	5.0 (2.5)	6 (2/7)	
Total score		54	70	36	64	69	50	50	56.1 (12.3)	54 (50/69)	
Mean (SD)		4.5 (0.9)	5.8 (1.2)	3 (2.4)	5.3 (1.3)	5.8 (2.4)	4.2 (2.0)	4.2 (2.0)			
Q_1_		4	5	1	4	4.8	2.3	2.3			
Median		5	6	2	6	7	4	5			
Q_3_		5	7	5.5	6	7	5.8	6			

^a^Patient

^b^Quartile 1=Q_0.25_; Quartile 3=Q_0.75_

^c^Positive statements: 1=strongly disagree / 7=strongly agree; Negative statements: 1=strongly agree / 7=strongly disagree

**Table 2:** Postintervention therapists’ TAM questionnaire responses^a^.

Statement		T1^b^	T2	T3	T4	T5	T6	T7	T8	T9	T10	T11	T12	Mean (SD)	Q_1_^c^	Median	Q_3_^d^	Mean (SD)
**Perceived user-friendliness**																		
	a)	6	5	6	7	7	5	5	5	5	6	6	4	5.6 (0.9)	5	5.5	6	4.9 (1.4)
	b)	5	6	7	7	7	4	5	5	4	5	3	4	5.2 (1.3)	4	5	6.8	
	c)	5	4	6	5	7	6	4	4	2	2	2	4	4.3 (1.7)	2.5	4	5.8	
	d)	5	5	7	5	7	6	4	4	4	3	3	3	4.7 (1.4)	3.3	4.5	5.8	
**Attitude towards using the exergames**																		
	e)	5	4	4	4	3	4	4	3	3	4	2	6	3.8 (1.0)	3	4	4	3.6 (1.4)
	f)	4	3	2	4	2	4	3	4	4	3	4	3	3.3 (0.8)	3	3.5	4	
	g)	7	5	7	3	3	5	4	6	3	2	6	5	4.7 (1.7)	3	5	6	
	h)	1	3	1	1	2	3	1	4	1	3	2	3	2.1 (1.1)	1	2	3	
	i)	4	5	3	3	2	4	3	4	4	4	5	5	3.8 (0.9)	3	4	4.8	
**Intention to use the exergames in the future**																		
	j)	2	3	1	2	2	2	2	3	3	4	2	3	2.4 (0.8)	2	2	3	3.7 (1.8)
	k)	7	6	7	7	6	7	6	5	6	5	5	5	6.0 (0.9)	5	6	7	
	l)	2	4	2	2	2	4	2	3	3	4	2	3	2.8 (0.9)	2	2.5	3.8	
Total scores		53	53	53	50	50	54	43	50	42	45	42	48	48.6 (4.5)	42.3	50	51	
Mean (SD)		4.4 (1.9)	4.4 (1.1)	4.4 (2.5)	4.2 (2.1)	4.1 (2.4)	4.5 (1.4)	3.9 (1.4)	4.2 (0.9)	3.5 (1.3)	3.8 (1.2)	3.5 (1.6)	4.0 (1.0)					
Q_1_		2.5	3.25	2	2.25	2	4	2.25	3.25	3	3	2	3					
Median		5	4.5	5	4	3	4	4	4	3.5	4	3	4					
Q_3_		5.75	5	7	6.5	7	5.75	4.75	5	4	4.75	5	5					

^a^Positive statements: 1=strongly disagree / 7=strongly agree; Negative statements: 1=strongly agree / 7=strongly disagree. Statements: Perceived user-friendliness a) The exergames manual was clear and understandable. b) I was easily able to train my patients for using the exergames. c) Learning to use the exergames independently was easy for my patients. d) I experienced learning to use the exergames as easy. Attitude towards using the exergames e) I generally have a positive attitude towards using the exergames. f) The exergames were a gain for my patients. g) The exergames were an unnecessary burden for my patients. h) The exergames were a relief of responsibility for me. i) The supervision of my patients was a pleasure for me. Intention to use the exergames in the future j) I can imagine using the exergames regularly as a training for my patients. k) I generally believe that my workplace supports the use of VR training possibilities for my patients. l) I would recommend using the exergames to other colleagues. ^b^T=Therapist ^c^Quartile 1=Q_0.25_ ^d^Quartile 3=Q_0.75_

### Perceived User-Friendliness

This subcategory was the most positively judged among patients with a mean 5.6 (SD 1.4) points and therapists with a mean 4.9 (SD 1.4). All patients agreed on the clarity of the manual and the ease of use of the exergames, while learning to use the exergames independently would not have been easy for patients 1 and 6 (P1 and P6; see Table 1). A possible explanation was the poor general computer knowledge that both patients identified. Thus there were associated uncertainties of what to do when sudden difficulties arose while playing, as stated by P6: “Sometimes I had difficulties in discharging the apples, and when I wasn’t able to keep them steady over the basket, then the apples refused to drop into the basket” (P6). P2 had the same experience in another game with the “faulty pieces”:

Yes, they [the exergames] sometimes weren’t technically well set. You touched it [the fruit], but it didn’t stick [to the virtual hand]

P2

To solve those difficulties in game control, P6 and P2 indicated that they contacted the supervising therapist for help. Using the Novint Falcon as haptic device to play the games was perceived as good by all patients. Patient P3 described that the device “acted up” now and then (not running smoothly or skidding of the whole device while playing), suggesting use of the mouse instead of the Novint Falcon as input device in order to solve this problem.

**Figure 4:**
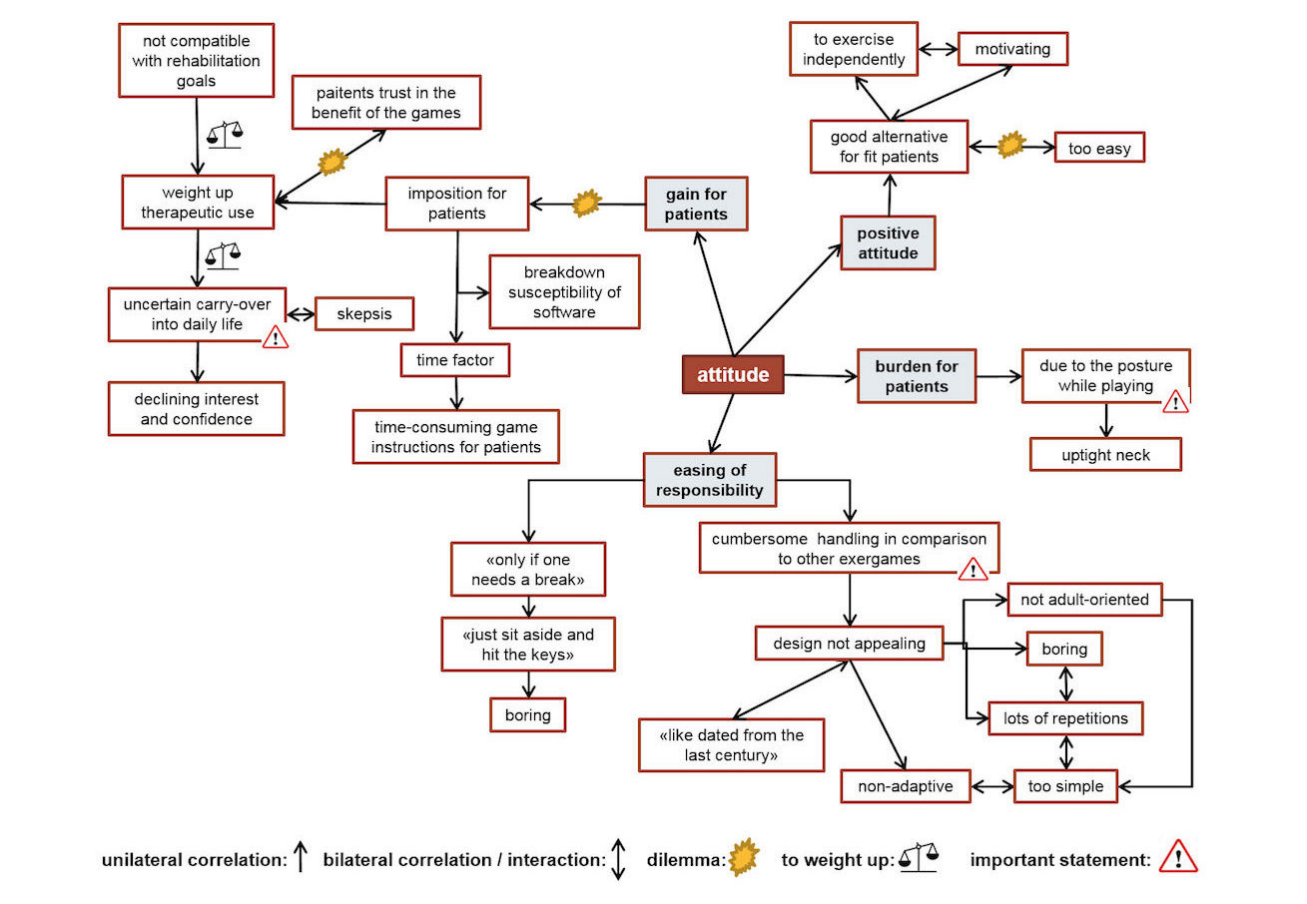
Focus group illustration map: attitude toward using the exergames.

The therapists all agreed on the game manual’s good understandability (Figure 3). They rated themselves as being capable of introducing the exergames to their patients (Table 2). However, they also stated that only the fitter patients were able to use the exergame station independently. For the more severely affected ones, guidance of the arm was necessary to play the games. This was experienced as no relief of responsibility for the therapists, which negatively influenced their attitude towards using the exergames (Figure 4). They further reported that some patients had difficulty understanding the purpose of the games. Therapists’ reasons for this were given as being either (1) due to patients’ poor self-awareness of their actual skills, making it difficult for them to explain to the patients the necessity of exercising their visuospatial exploration skills, or (2) due to the game design, which was experienced as being unappealing by both patients and therapists in combination with the abstract game control. The breakdown susceptibility of the software and the suboptimal posture, especially the use of the chin rest, were major critique points mentioned by most therapists (Figure 3). The former explained why therapists reported experiencing loss of valuable therapy time, as they often had to re-boot the system, keeping patients waiting while the computer restarted. Thus the exergames were rated as being somewhat impractical, although therapists recognized that they were testing in a pilot phase.

### Attitude Toward Using the Exergames

The general attitude of the patients toward the use of the exergames was 4.8 (SD 1.9) points (Table 1). The therapists rated it a mean total score of 3.6 (SD 1.4) points (Table 2). The patients experienced exercising with the games to be motivating and interesting and also as a “welcome change" to the conventional therapy methods provided in the Rehabilitation clinics: “Those games were a welcome diversion to normal neuropsychology, where you just sit face-to-face and have to do exhausting things all the time” (P5).

However, most patients preferred the conventional therapy methods over the exergames intervention, as they described it difficult to understand the purpose of the games:

In the beginning, I didn’t really understand what all this meant; for what the games were good for. Then they [the therapists] explained it to me. After they had told me what aspects I had to pay attention to, then it was all good.

P2

I couldn’t make sense out of it [the exergames]. I always had the feeling that the things there—those tests—were meant for ones with very severe brain damage, who weren’t back on their feet yet. […] But not for me—I don’t have such severe damage!

P3

I don’t know what they [the exergames] would have been useful for. And no matter how much you have scored, you couldn’t see the progress you had made.

P6

The patients experienced conventional methods as more effective than the VR intervention:

I didn’t have the impression that it [the exergames intervention] did yield much. I experienced it as being a bit silly. I had the impression that the other things simply helped me much, much more. […] The eyes were not equally challenged to move back and forth.

P6

The content of the games was judged as “not bad” (P4). P4 perceived the exergame “puzzle” as being difficult because the puzzle template was displayed only once at the beginning, requiring the player to piece together the puzzle out of memory. The speed of the games and the related short reaction time was another difficulty mentioned by most patients as being experienced during play. They described being initially very motivated to play the games, but over the course of the 3-week intervention, their enthusiasm decreased, as they started to perceive playing the games as “boring” (P1, P4) and even “childish” (P1, P3):

You know, piling the ABC can be done by a first- or second-former! And to burst balloons that pop up out of a hole isn’t very demanding either” (P3); and “Boring! In the beginning, it was good. But most recently…it was complicated to look through this thing [chin rest], you know. The other games they had were more interesting in a way.

P4

Using the chin rest while playing was the main reason why most patients experienced the exergames as exhausting (mean 3.9 [SD 2.3]):

This [chin rest] wasn’t useful! I couldn’t sit in an upright position and look through [the chin rest] to scan the whole computer screen. This was exhausting. You also weren’t able to turn your head.

P4

The suboptimal posture of the patients while using the exergames was also problematic for the therapists (Figures 3 and 4). It was the main reason why some therapists rated them as an unnecessary burden. They described observing their patients sitting tilted to the left side in their wheelchair due to their VSN symptoms, watching past the chin rest instead of looking through it while playing. Furthermore, the therapists expressed reservations regarding the therapeutic use of the exergames. Those reservations were based on their uncertainty of achieving a carryover effect of visuospatial room exploration skills trained in a virtual environment into the real world. Additionally, they had difficulties in perceiving the VR intervention as supportive to achieve the patient’s rehabilitation goal, namely to regain independence in daily life as well as possible. The therapists further described that their patients fully trusted them in the choice of therapy intervention to improve their skills (Figure 4). This blind trust gave the therapists a dilemma: on the one hand, they wished to use conventional therapy methods instead which they knew to be effective, but on the other hand, they recognized that the patients had agreed (and were eager) to participate in the study using this novel intervention. Despite this rather negative attitude towards exergames use, some therapists rated them as being a motivating alternative for fit patients to exercise independently, although they also rated them as being too easy for some patients.

### Intention to Use the Exergames in the Future

Using the exergames regularly in the future was not viewed as conceivable yet, either among patients (mean 3.9 [SD 2.1] points) or therapists (mean 3.7 [SD 1.8] points). Most patients perceived the exergames as a good pastime and diversion that helped shorten the long days in the rehabilitation clinic (P4, P6, P7) but indicated that they would prefer doing activities other than gaming once back at home: “Up there [in the rehabilitation clinic], I thought that it is way better to do this [playing the exergames] than lying in bed or sitting on a chair while doing nothing” (P6). P5 and P7 described themselves as not being “a computer freak” (P7) or “a gamer” (P5) and therefore not wanting to use the games further at home. P3 missed the relevance to real life of the games, making the following suggestion for improvements:

Well, maybe tests that are more related to practice. You know, where you see: “Ah, this could be useful!” […] For example doing an exercise you will need in the future when you want to drive a car again. Reaction or such things…which will help me to go ahead.

P3

For some patients, the games could have been more challenging and entertaining. P7 did not experience much pleasure while playing:

Not really…well, when I was successful, then I felt pleasure anyhow. Then I thought: ‘Indeed, I am not as dull as I thought!’ […] It simply worked out somehow, but not as good that I would have felt pleasure to play more.

P7

Remarkably, most patients were nevertheless convinced that their family and friends would support them using the exergames at home. The patients also stated that they would recommend the exergames to other patients (Table 1). Reasons might be that the support of their relatives is taken for granted—no matter what they were doing to get better—and that they believe that trying everything to get better is the best rehabilitation strategy, including novel therapy methods like the exergames: “One should leave nothing undone, and try out everything!” (P7).

**Figure 5:**
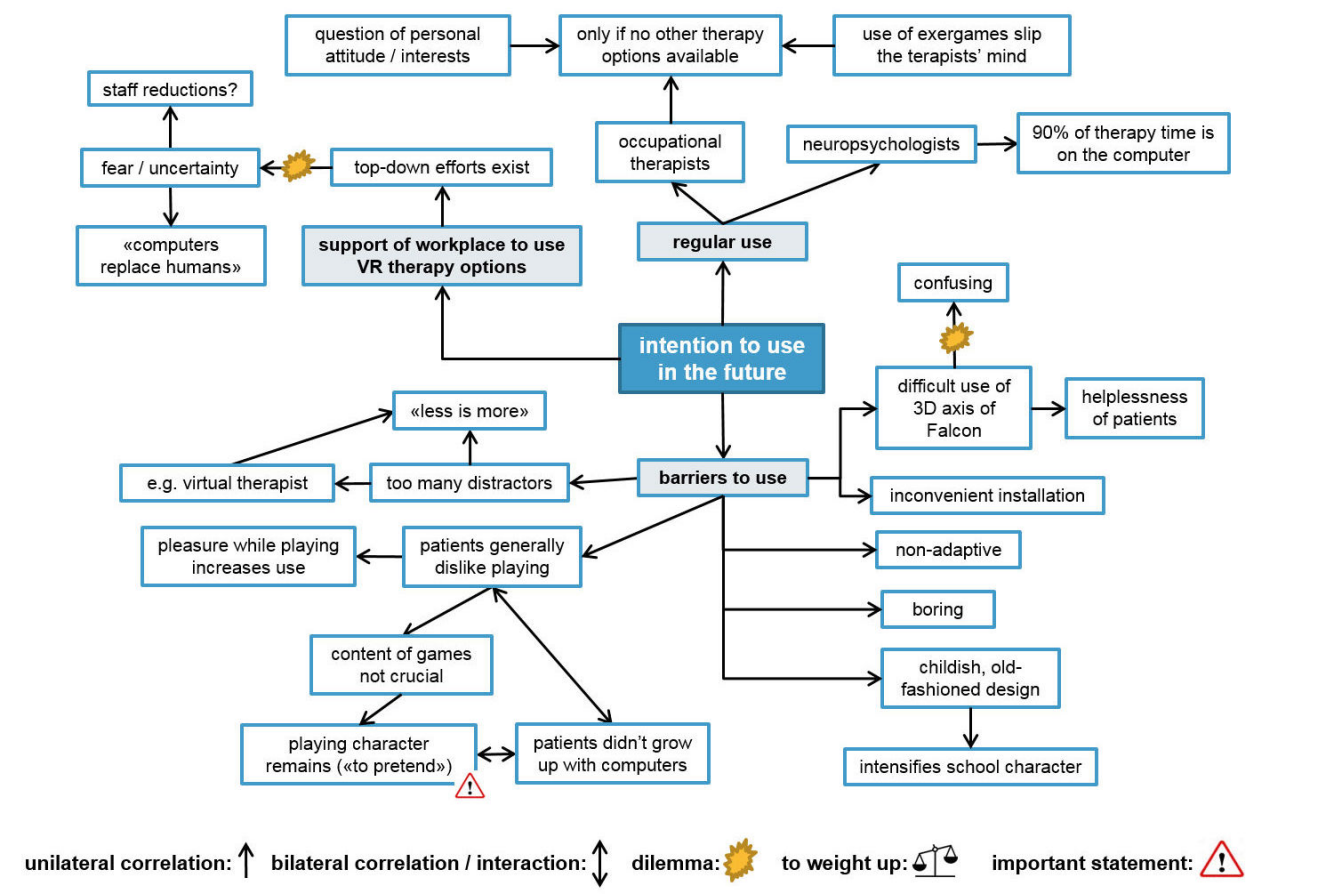
Focus group illustration map: intention to use the exergames in the future.

The therapists were not yet ready either to use the exergames in the future or to recommend their use to other colleagues—at least in the version used for this study—although all therapists were convinced that their workplace supports the use of VR training methods (Table 2). The neuropsychologists in particular were experienced in using the computer as a means of therapy and therefore accustomed to high-tech VR methods. The OTs, however, were rather restrained towards VR methods, some even fearful of being replaced by computers in the future (Figure 5). Barriers to future use of exergames were diverse and numerous; for example, the benefits of virtual versus equivalent real-life tasks was mentioned by the OTs, who expressed preference for the latter therapy option. The nonadaptiveness of the software was another barrier highlighted by the neuropsychologists, as they were used to exergames with this feature.

**Figure 6:**
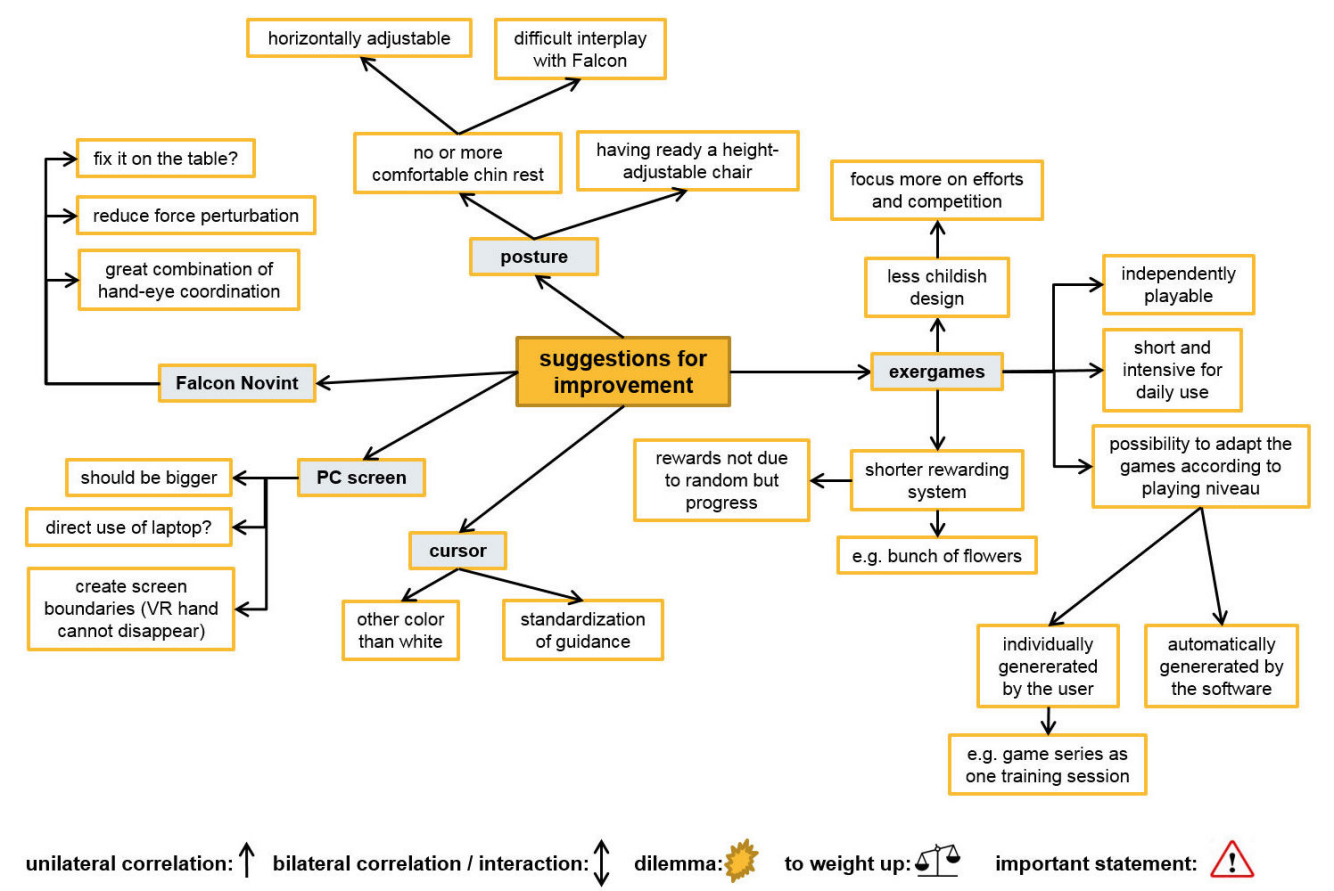
Focus group illustration map: suggestions for improvements of the game-based virtual reality intervention.

The therapists proposed suggestions for improvements for all mentioned barriers (see Figure 6), which, given that those improvements are implemented in a new version of the exergames, indicates that a new version of those exergames would be used in the future.

## Discussion

### Principal Findings

This usability study aimed to quantitatively and qualitatively assess user perspectives (patients and therapists) of using REWIRE exergames as a novel rehabilitation intervention to treat VSN symptoms due to stroke. The findings showed that the patients as end users generally rated the use of the exergames more highly than did the therapists. Most patients experienced the games as motivating, interesting, and a welcome diversion in their daily routine during their inpatient stay in the rehabilitation clinic. The feeling of joy and motivation while playing was also described in other studies assessing user perspective in stroke patients testing a novel VR intervention [23,37,38]. Those studies tested games aiming to improve motor control in the affected arm due to hemiparesis following stroke without VSN symptoms. In our study, the patients controlled the games with a haptic device using their unaffected arm, in order to focus on improvement of cognitive skills. Most participants liked using the Novint Falcon instead of the mouse to control the games. However, some patients described having difficulties in grasping and releasing virtual objects. They confirmed that in one game, they had to touch an object with the index finger of the virtual hand to grasp it and in another game with the palm of the hand. This discrepancy was experienced as being misleading. In order to standardize the game control, Mainetti et al [39] suggest optimizing the degree of overlap between the virtual hand collision region and the target collision region.

Our sample, however, suffered from neglect and a certain related level of anosognosia [40]. They nevertheless experienced pleasure while playing. This is in line with other findings from a satisfaction questionnaire where stroke participants with neglect symptoms indicated enjoyment of the VR experience [19]. Despite having fun while playing, the presence of anosognosia in our sample negatively influenced their understanding of the purpose of the exergames. Inability to fully understand the purpose of the intervention was also a topic in a focus group interview with stroke patients without VSN symptoms [24]. It is important to make sure that patients understand the game purpose, so as to meet their expectations and to avoid frustration [23]. Although we paid weekly visits to participating rehabilitation clinics to discuss progress and progression of the exergaming with patients and therapists, it nevertheless seemed difficult for some of the former group to understand the purpose of the treatment strategy. This was particularly the case for the more severely affected patients.

**Figure 7:**
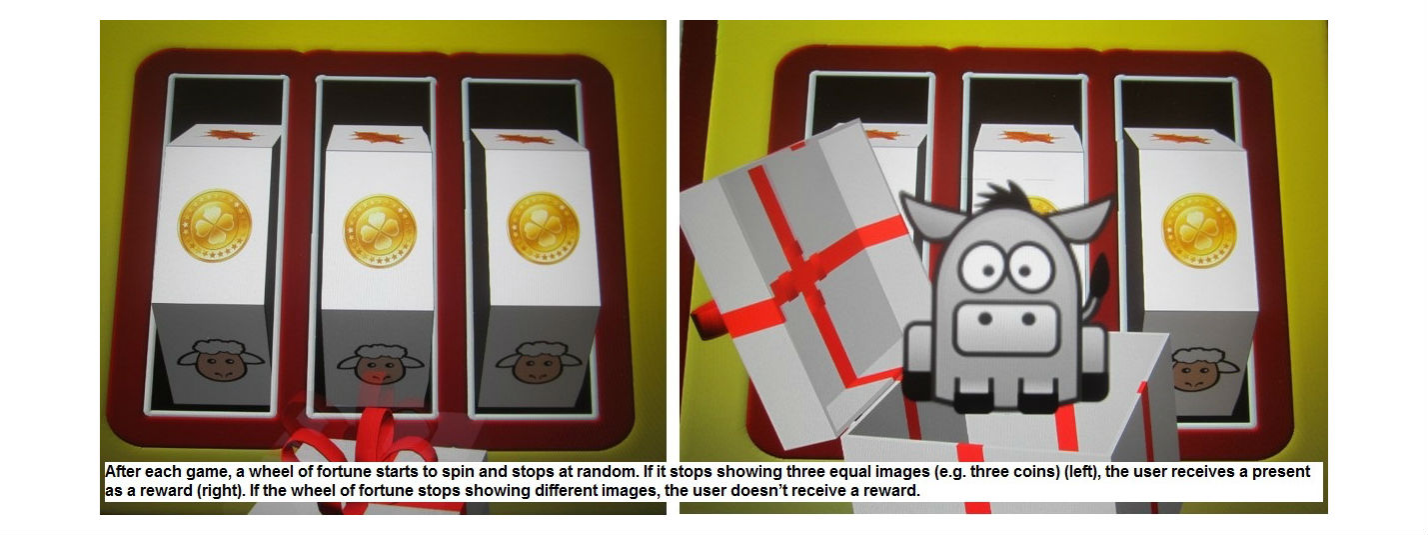
Rewarding system of REWIRE exergames.

Mainetti et al [39] tested exergames in a single patient with chronic stroke who had VSN symptoms. This patient liked the exergames and was not bored while playing them. Most of our patients, however, experienced a decreasing enthusiasm during the 3-week intervention and started to perceive the activity as boring, even though games regularly and individually progressed and were designed according to therapeutic principles [13]. It seems that basing the selection of games on personal interest of the patient could not enhance motivation while playing either. Paying attention to the diversity and progression of game complexity is no guarantee of constant use and engagement over time. Other studies testing different VR interventions with patients with cerebral palsy also described a reduction in engagement over time [41,42]. Therefore, reasons for this decreasing enthusiasm other than a suboptimal balance of providing a challenge while still enabling success might be the time point of the intervention and the lack of feedback in the achieved game scores. Compared to other stroke samples [24,25,37,39], our patients were still in the early stage of recovery and were still hospitalized, therefore in the situation of receiving daily therapy sessions with which they could compare the REWIRE exergames. In this context, it is perhaps understandable that the exergames—still a test version—fell behind other VR therapy options that are long-established in the market. Furthermore, testing a novel therapy option with stroke patients in their chronic stage, when regular therapy often might have stopped, evokes hope for further motor or cognitive improvements and therefore increases motivation [25]. Another reason for the decreasing interest might be seen in the fact that our games did not display the achieved results after each training session, unlike Lewis et al’s [25] submarine game, for example. Although this option was provided by the software, we had decided not to activate it, as the achieved scores after each game/session were not yet storable. This prevented the patients seeing progression over time. We were aware that being unable to see the achieved scores equaled a lack of feedback regarding the patient’s personal progress. However, positive feedback and measures of success are critical components to enhance engagement [23,24]. There was a rewarding system after each REWIRE game (Figure 7), but as this was random and not performance-based, patients did not care for it.

The lack of feedback experienced by fitter patients, combined with their perceptions of being insufficiently challenged while playing the exergames, might be reasons why they indicated preference for conventional therapy methods with a “real” therapist over this VR intervention. Their experiences of the games as “a good pastime and diversion” suggests that most patients did not see this as a rehabilitation intervention per se, supporting their preference for conventional therapy. This is in line with other findings, where stroke survivors experienced the novel games as supplementary to conventional therapy, the latter being viewed as providing beneficial rehabilitation [25]. Conversely, the majority of patients in another study reported experiencing VR interventions as useful as conventional therapy [43]. The nature of play that is inherent to games may be perceived differently among adult patients, as the therapeutic benefit may not be as obvious as during conventional therapy, also depending greatly on how the virtual environments were designed [23]. It might be that having prepared a predefined set of games to be played during several training sessions while only progressing difficulty levels within the same games—as it was suggested by some of the therapists—might have helped patients perceiving the exergames as a (repetitive) rehabilitation intervention. However, we preferred letting them choose and switch games according to their individual preferences to (1) keep motivation as high as possible and (2) give them the opportunity to test all exergames during the intervention.

The REWIRE game design was rated as having limited appeal by both therapists and patients. The therapists in particular wished to have games that would be self-adaptive to patient progress in order to experience ease of responsibility. For example, they missed the opportunity to prepare a series of games that would then automatically run through during an intervention session. The lack of facility within software for patients to save and return to previously achieved difficulty levels between sessions was also noted. Those features would allow the patient to start directly at the right difficulty level and subsequently play the game independently without the therapist needing to adjust the settings before and during the training session. Although the goal of such VR interventions is to create a game menu that patients can run themselves with little input from others, it is nonetheless imperative that an expert (ie, therapist) guides progression of the games to maintain the therapeutic basis of the intervention. In one study, where game speed and progression advanced automatically, the users were overwhelmed, which negatively influenced motivation and engagement in the game intervention [44].

Some of our patients indicated that they missed the exergames’ relevance to real-life tasks—feedback also given by other stroke patients testing similar interventions [25]. This is despite the fact that we had tried to design them to be as much alike as possible. Male patients in particular perceived being able to drive a car as very important to them and therefore wished to be able to train those driving skills on the computer. Such conflicts with real-life expectations have also been described by Lewis and Rosie [23], suggesting a selection of environments that are deliberately unreal. Such simple environments have the advantage of avoiding unnecessary distractors by providing a restricted amount of stimulation, thus targeting the required rehabilitation effect. On the other hand, they comprise a risk of boredom and a related reduction in engagement for both patients and therapists.

When supporting patients to play the REWIRE exergames, some OTs expressed uncertainty in achieving carryover effects into real-life tasks. This uncertainty was one of the reasons why they would have preferred to use time for the training of real ADL rather than game play to achieve the rehabilitation goals set for their patients. Indeed, evidence for positive carryover effects of VR interventions into real life is limited [45,46]. For example, Gruskin et al [46] observed increased awareness of the involved extremity as well as greater carryover into ADL when using an auditory feedback device to alert a patient with left hemineglect when his flaccid upper extremity was in a dependent position. Gates et al [45] compared walking overground and on a treadmill surrounded by a virtual environment that applied optic flow in individuals with and without transtibial amputation. They found that both groups walked with similar overall kinematics (eg, knee flexion/extension) and kinematic variability (ankle, knee, or hip) on the treadmill as they did overground. Their results suggest that treadmill training in a virtual environment should be sufficiently similar to overground walking in the real world that changes carry over.

Further reasons why the therapists would have preferred use of rehabilitation time for conventional therapy rather than for testing the novel VR intervention was the breakdown susceptibility of the software—giving them the feeling of wasting too much therapy time. Additionally, the use of the chin rest forced the patients to sit in a nonergonomic posture. This posture was also the reason why many patients got tired while playing, rather than because of cognitive challenge. When planning this study, we did not expect the chin rest to be a major problem when playing the exergames. Its use was precipitated by a need to avoid compensatory movements of the head. However, according to the feedback of all participants, the use of the chin rest for a whole therapy session of approximately 30 minutes was too exhausting. We therefore recommend the use of a chin rest for short assessments only rather than for a whole therapy session [47]. Such technology limitations have also been described in other studies testing VR interventions [42,48]. For example, Wille et al [48] found a correlation between software failures and reduced ratings of fun while playing. Li et al [42] have described difficulties in positioning patients with postural impairments so that they were able to operate the VR system. Not surprisingly, such technology limitations are associated with negative feedback from the users, as was the case in our sample. Those limitations were also determinative of participants’ ratings of limited intention to use exergames in the future. Other perceived barriers were not being a “gamer” (patients), as well as the fear of being replaced by computers in the future (OTs). The neuropsychologists did not share this fear, as they were more used to computer-based interventions than the OTs. As a consequence, the neuropsychologists as computer experts were the most critical users of our exergames.

### Limitations

Some limitations of this study should be discussed. First, most of the stroke patients needed assistance in completion of the TAM questionnaire, either in retaining the paper-based questionnaire while it was on the table due to their hemiparesis, or in being helped to read the questions due to their VSN symptoms. Both of these issues may have influenced their responses. A touch-screen version on a tablet fixed on a table to avoid side slipping for those stroke patients who suffer from hemiparesis would allow questionnaire completion with one hand only. A button placed on the right margin of the tablet could be designed to audio-display the questions, making reading of the questions unnecessary. Second, the fact that the therapists participating in the focus group were working colleagues from the same team might also have influenced their interactions and utterances during the interview. For example, in both interview groups, the team leader was also present, which might have inhibited some participants in expressing what they really thought about the exergames. We therefore chose focus group illustration maps for data analysis. Together with the flip chart notes taken directly during the interview, those FIMs allowed a precise summary of the group statements without exposing someone through using quotes, where they might recognize the person who had said that. Third, the fact that the main researcher (BC-T-A) knew all participants quite well at the time point of the interview influenced her way of conducting the individual and focus group interviews. Holding preunderstanding about the patients’ life from former meetings during data acquisition for the feasibility study might have influenced her way of formulating questions differently than when she would have met the patient for the first time. However, the interview quality probably had improved thanks to the already established relationship. Being an occupational therapist like half of the participating therapists further influenced the flow and conduct of the focus group interviews. However, speaking the same professional language might have facilitated formulating experiences made with the patients and exergames. Fourth, the recruitment of stroke patients with VSN symptoms in a clinical setting who were fit enough to test the game-based VR intervention was quite difficult. Testing such an intervention in a later, chronic stage where most patients are in a better health condition might have been easier. However, all patients were excited to take part in a research project during their inpatient stay and they cherished being asked for their personal opinion not only in a questionnaire, but also in a face-to-face interview.

### Future Work

Lewis and Rosie [23] were entirely correct in their statement that “it may appear impossible to design a system that appeals to all users” (p. 1884). However, we should not overlook the fact that, despite all the critiques mentioned by users, most patients enjoyed playing the exergames. The criticisms identified are a motivator to improve the existing game design in order to achieve an optimal rehabilitation effect. Therefore, before thinking about testing the REWIRE exergames in a larger controlled trial of stroke patients with VSN, for example, the game design should first be modified according to the suggested improvements. Decisions should be made regarding the degree of realism of the virtual environments: should we design environments as unreal as possible [23], or as real as possible by using a tool such as Google Street View [19], for example? Immediate feedback of the achieved game scores should be implemented together with a graphical overview of the changes over time to enhance engagement and motivation. The flexibility of the software should be increased, for example, by creating a function to save the chosen difficulty level for each game. Future work could examine if the frequency and time of game play—in our study on a daily basis over 3 weeks—or if providing a predefined set of exergames to be played instead of having free choice of game selection, influences user perspectives on the exergames. Results have shown that the use of a chin rest to control compensatory movements of the head is not recommended for a whole therapy session. Furthermore, evidence is needed to explore possible carryover effects of such VR interventions into real life in order to enhance acceptance of such interventions among therapists.

### Conclusion

This study provided insight into user perspectives based on quantitative and qualitative statements of stroke patients suffering from VSN and therapists using novel exergames to explore the hemineglected left space in an inpatient setting. The results showed that all users perceived the REWIRE exergames as user-friendly, but that they would not necessarily entertain their use in their current format. The general attitude toward using the exergames was more positive among the patients than among the therapists. Recommendations for improvements of the exergames were mainly formulated by the therapists. Feedback suggests that once those recommendations could be realized, then the REWIRE exergames intervention could be explored using further trials. It is therefore of the utmost importance that end users (patients) and experts (therapists) are involved in order to achieve acceptable and user-friendly VR game-based rehabilitation methods.

## Appendix

Multimedia Appendix 1 REWIRE VSN exergames: Short instructions for the patient.

## Abbreviations

ADL: activities of daily living
CBS: Catherine Bergego Scale
FIM: Focus group Illustration Map
OT: occupational therapist
RBL: right-sided brain lesion
REWIRE: Rehabilitative Wayout in Responsive Home Environments
TAM: Technology Acceptance Model
VR: virtual reality
VSN: visuospatial neglect
