# Supplementary material for: User Perspectives on Exergames Designed to Explore the Hemineglected Space for Stroke Patients With Visuospatial Neglect: Usability Study
Source: JMIR Serious Games. 2017 Aug 25;5(3):e18. doi: 10.2196/games.8013 (PMC5591406; doi:10.2196/games.8013)
Supplement: Multimedia Appendix 1 [file games_v5i3e18_app1.pdf]

**Bubbles**<br>Stir all the bubbles with the stick. Only stir those matching the dragon’s color.	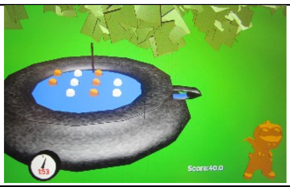
**Balloon Popper**<br>Pop the red and yellow balloons. Avoid the dark blue ones.	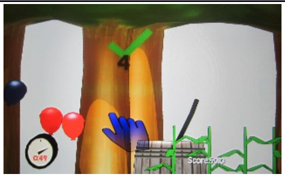
**Mix Soup**<br>Take all ingredients from the shelf on the left according to the recipe on the right and put them in the saucepan.	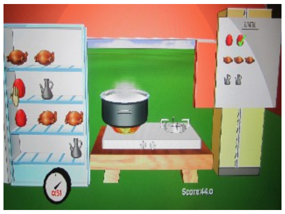
**Gathering Apples**<br>Pick all apples from the tree and put them inside the basket.	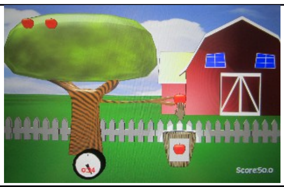
**Faulty Pieces**<br>Pick all fruits displayed on the computer screen from the assembly line and put them in the basket.	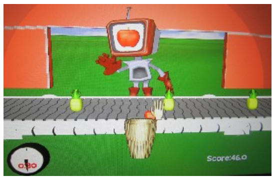
**Shape Follower**<br>Clean the dirty path around the house. Remember how many raccoons crossed your way.	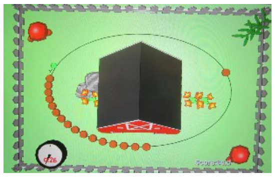
**Sequence Builder**<br>Build the right sequence of hay bales: 1, 2, 3, … or A, B, C, ….	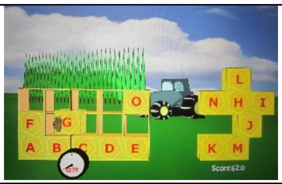
**Puzzle**<br>Put together the puzzle.	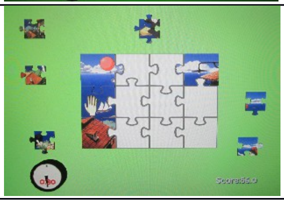
**Dog Leash**<br>Take your dog for a walk. Let him collect the frisbees on the way, but avoid the trees, stones and the chicken crossing the path.	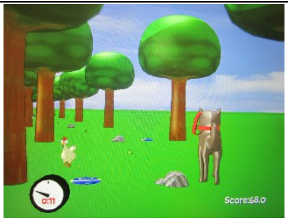
